# Supplementary material for: NEDDylation promotes nuclear protein aggregation and protects the Ubiquitin Proteasome System upon proteotoxic stress
Source: Nat Commun. 2018 Oct 22;9:4376. doi: 10.1038/s41467-018-06365-0 (PMC6197266; doi:10.1038/s41467-018-06365-0)

NEDDylation promotes nuclear protein aggregation and protects the Ubiquitin Proteasome System upon proteotoxic stress

Maghames et al.

Supplementary Information

Supplementary Figure 1

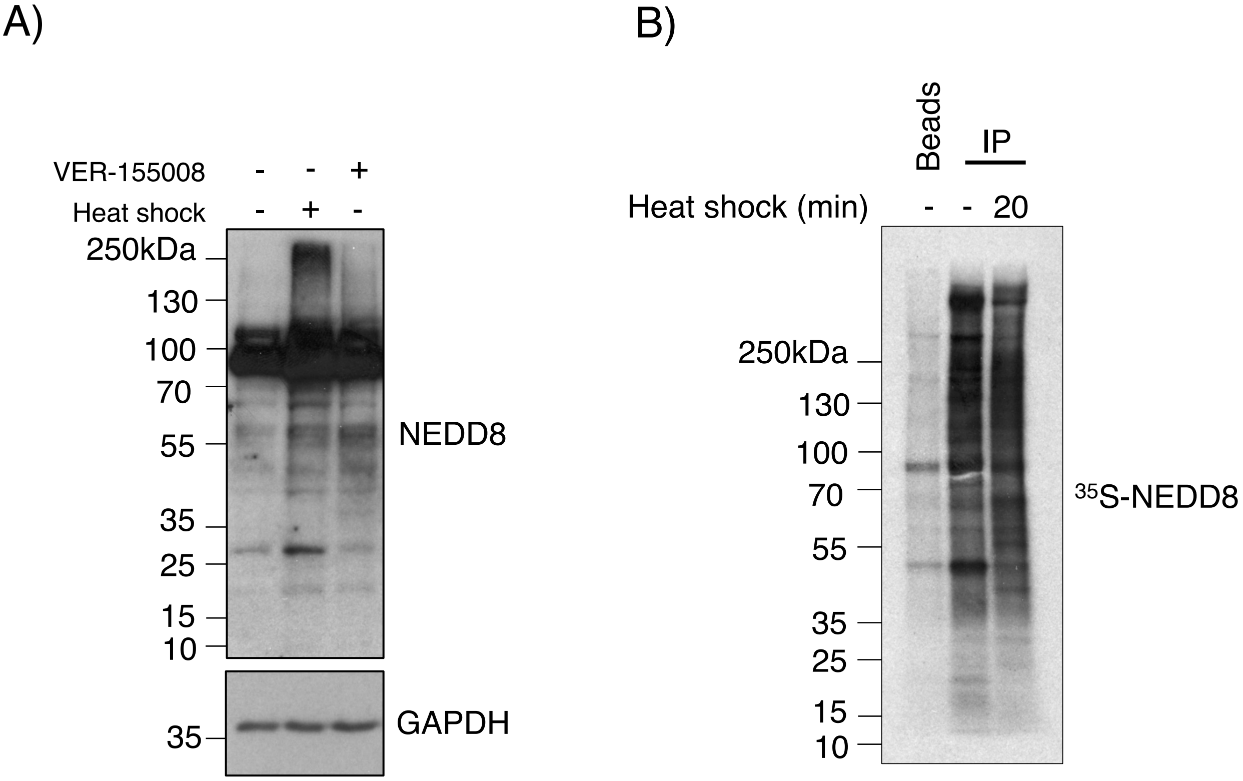

Supplementary Figure 1. Characterization of the NEDD8 response to proteotoxic stress. A) U2OS cells were either untreated, heat shocked for 30min at 43°C or treated with the HSP70 inhibitor VER-155008 at 100μM for 5hrs. Cells were harvested, lysed in 2xSDS lysis buffer and extracts were analysed by western blotting as indicated. B) U2OS cells labelled with ^{35}S-Methionine were either untreated or heat shocked at 43°C for 20min. Extracts were used for immunoprecipitations with anti-NEDD8 antibody and immunoprecipitates were analysed by SDS-PAGE. Gels were dried and exposed to X-ray film.

Supplementary Figure 2

A)

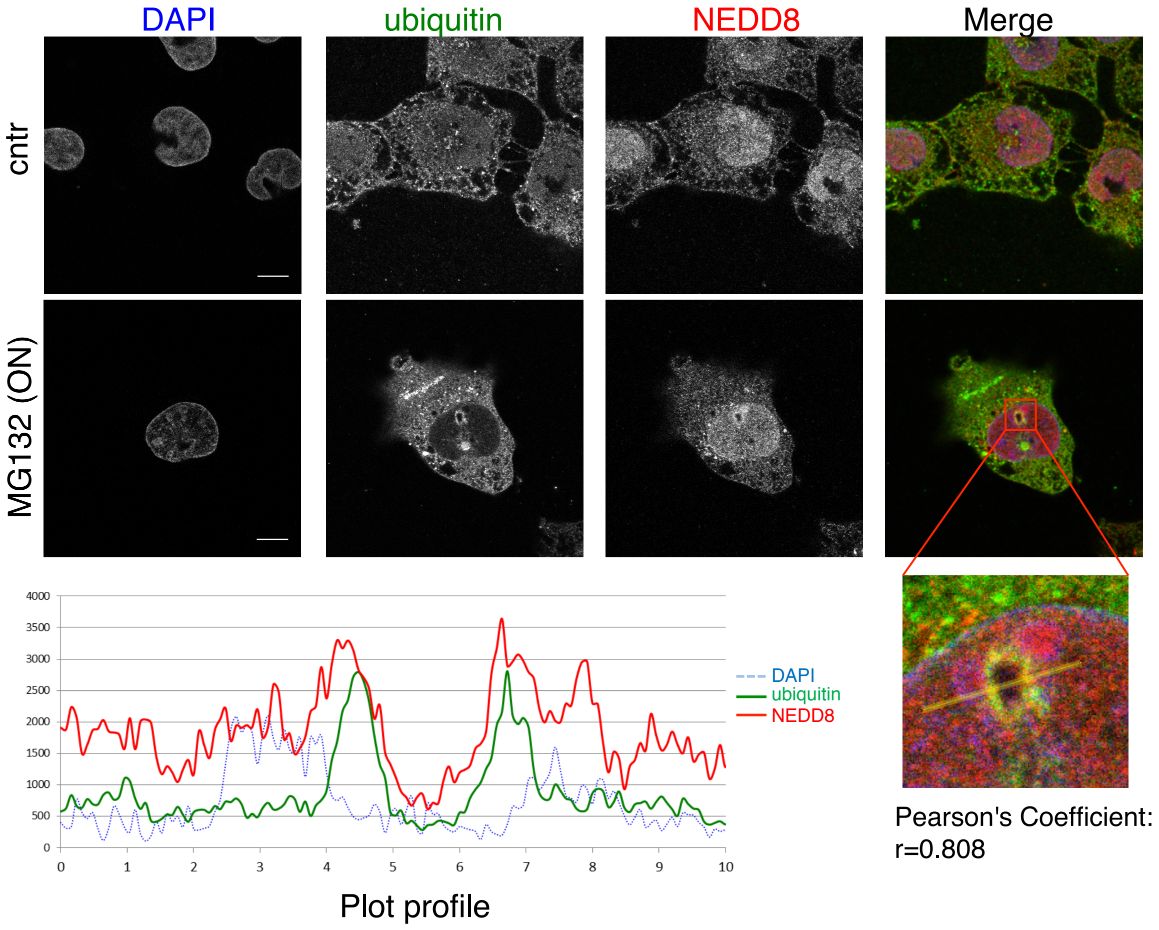

Supplementary Figure 2

B)

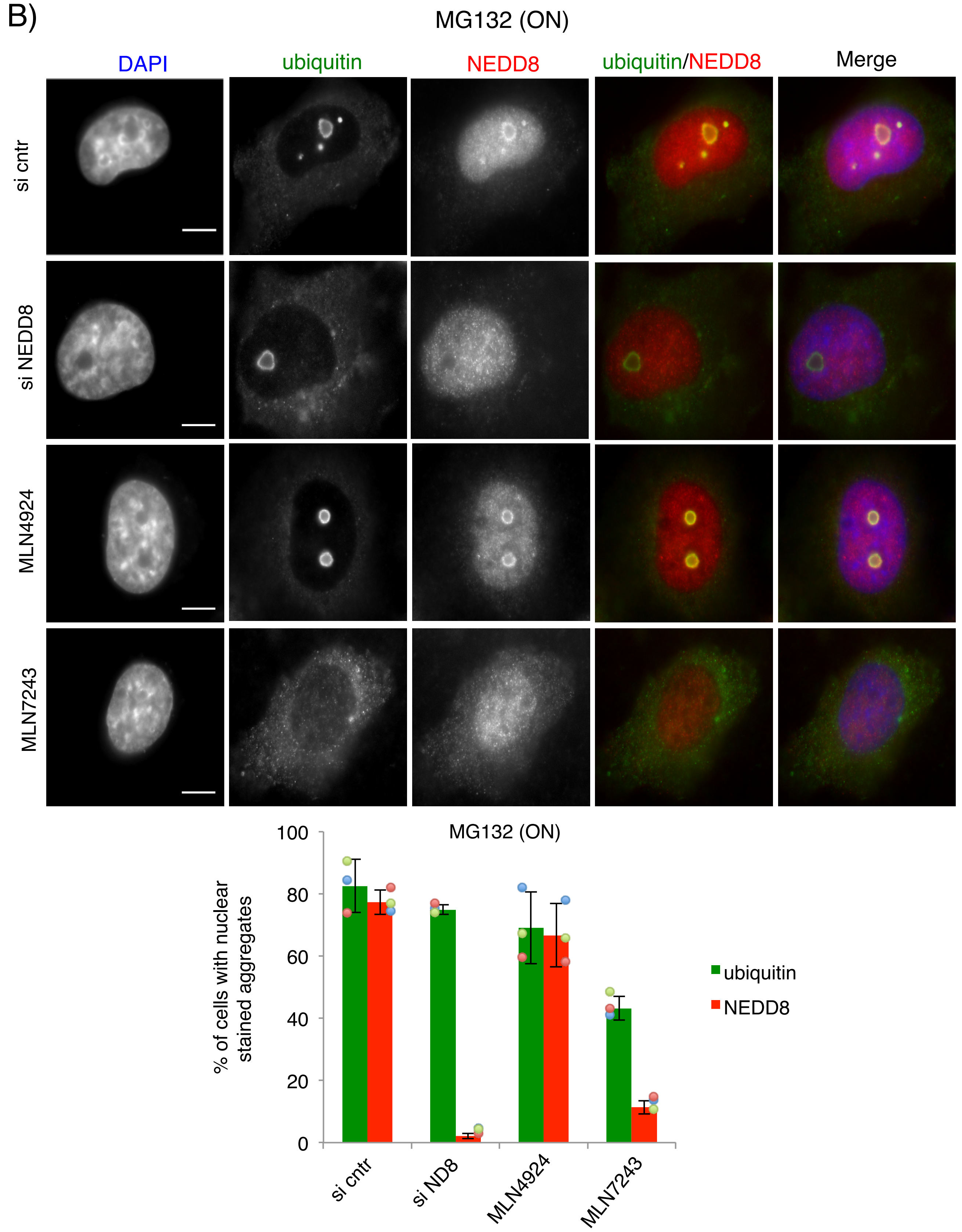

Supplementary Figure 2

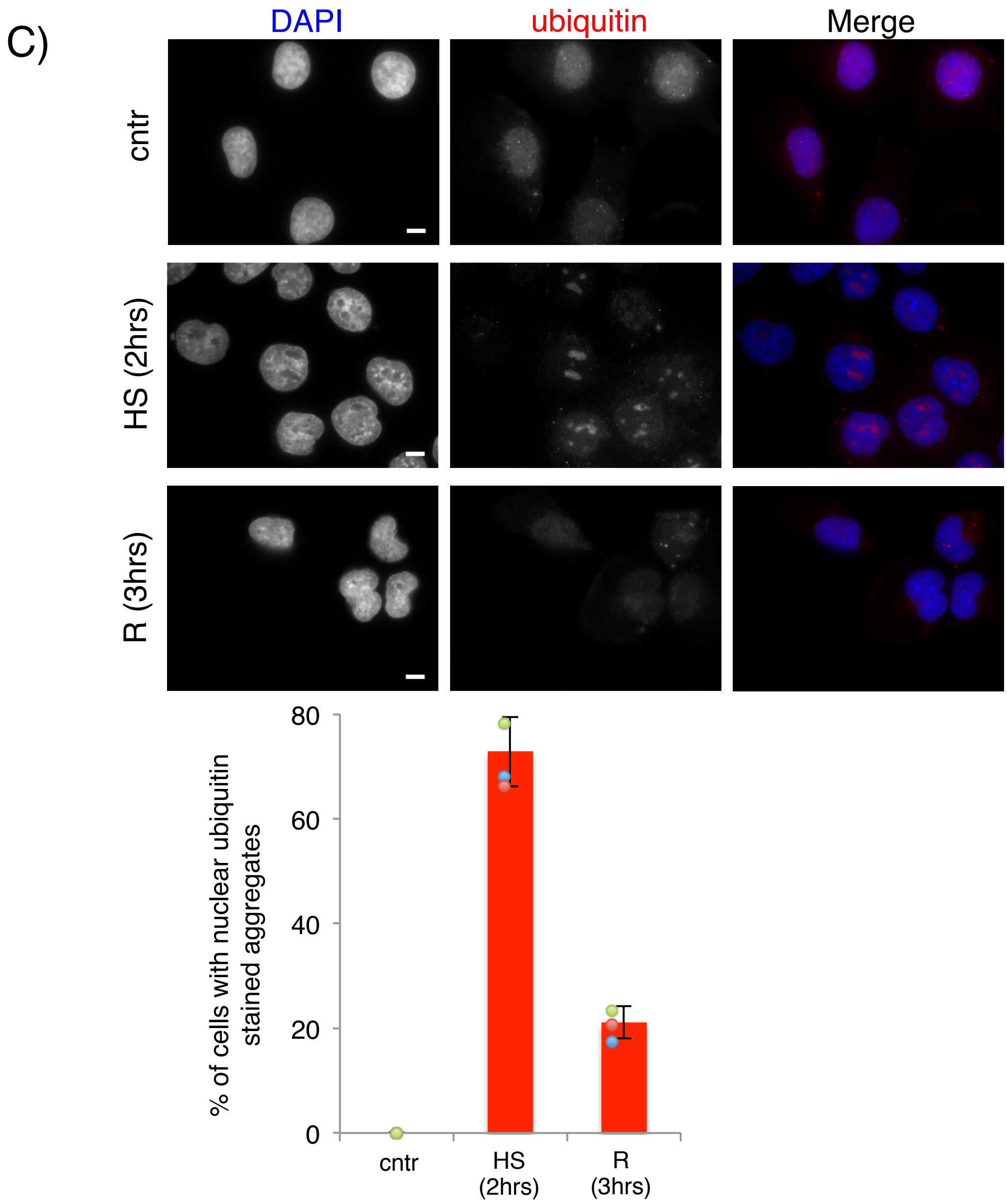

Supplementary Figure 2. Co-localisation of NEDD8 with ubiquitin in nuclear aggregates upon proteotoxic stress. A) Similar experiment as in Figure 1E. The co-localization between NEDD8 and ubiquitin in the nuclear structures was analysed as described in methods. The plot profile represents the NEDD8/ubiquitin signal in the presented single cell. B) Experiment performed as in Figure 1F except cells were treated with MG132 (5µM, overnight). Quantitation represents the average of 3 independent experiments +/- SD. Approximately 100 cells were used for each condition. C) H1299 cells were heat shocked and then allowed to recover at 37°C as indicated. Cells were stained with anti-ubiquitin antibody and DAPI. The bottom graph is the quantification of the experiment in (C). Data represent the average of 3 independent experiments +/- SD. Approximately 100 cells were counted per condition. Scale bars, 10µm.

Supplementary Figure 3

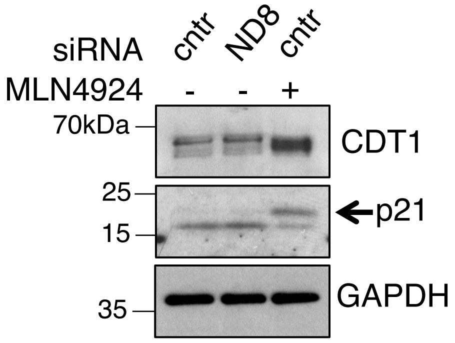

Supplementary Figure 3. NEDD8 knockdown does not affect CRLs activity. Extracts from experiment performed in Figure 2D were analysed by western blotting for the indicated proteins.

Supplementary Figure 4

A)

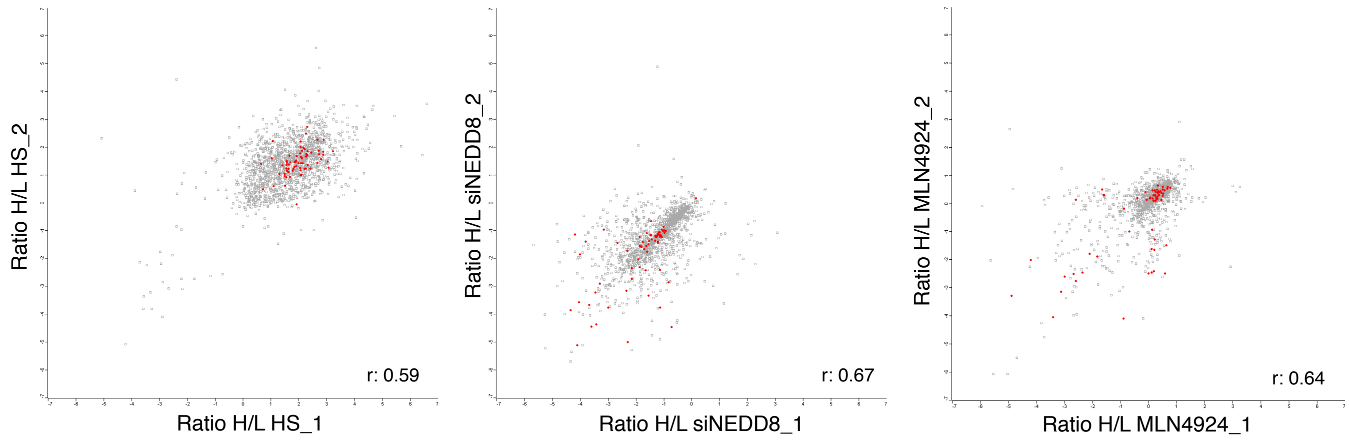

B)

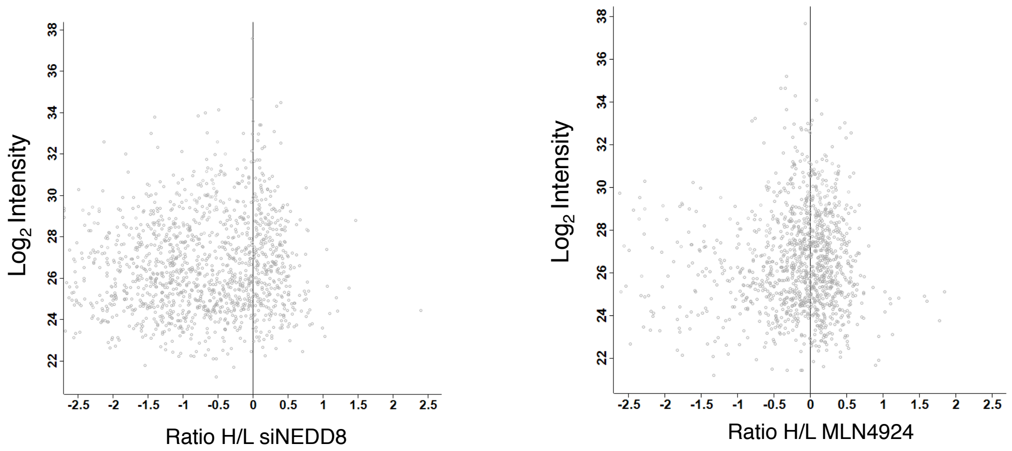

C)

Increased aggregation (siNEDD8 specific)		
Pathway description	Gene count	False discovery rate
rRNA processing	5	0.00364
RNA processing	8	0.00364
Ribosome biogenesis	5	0.00768
Nucleic acid metabolic process	15	0.00768
RNA metabolic process	14	0.00946

Supplementary Figure 4. SILAC proteomics on heat shock induced aggregates. A) Scatter plots between two replicate experiments performed for each condition, including the Pearson correlation coefficient. In red is the distribution for ribosomal proteins. B) Scatter plots of the mean values of two replicate experiments for siNEDD8 and MLN4924. C) Table represents the pathways for which proteins had an increased heat shock-induced aggregation specifically upon NEDD8 knockdown but not by MLN4924.

Supplementary Figure 5

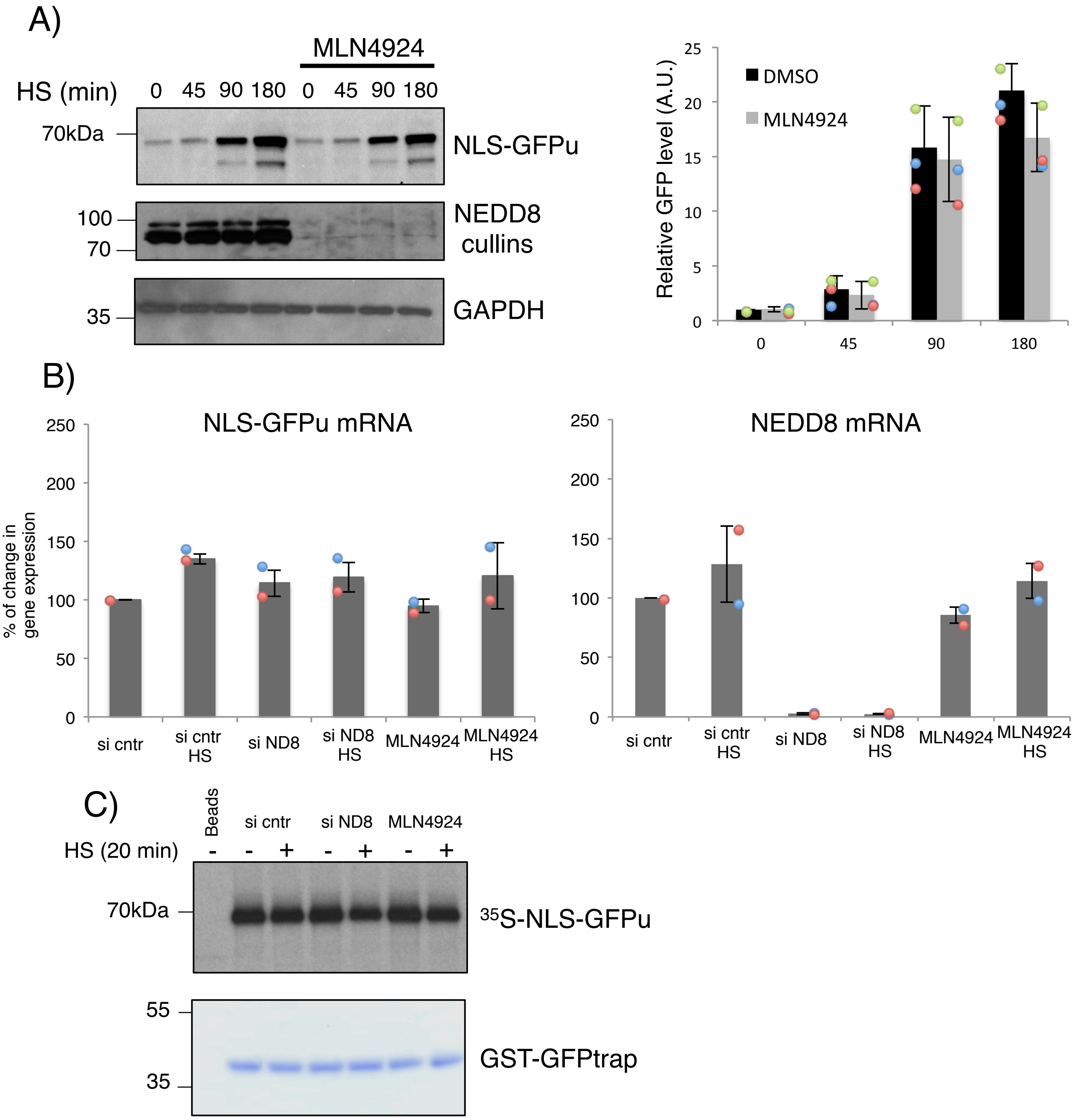

Supplementary Figure 5. Effect of MLN4924 treatment and NEDD8 knockdown on protein levels, mRNA expression and protein synthesis of NLS-GFPu. A) HEK293 cells stably expressing NLS-GFPu were either untreated (DMSO) or treated with MLN4924 (200nM, 48hrs) before heat shock as indicated. Extracts were used for western blot analysis for the indicated proteins. Graph on the right represents the average of 3 independent experiments +/- SD. B) HEK293 NLS-GFPu cells were transfected and MLN4924 treated as indicated before exposed to HS for 20min. mRNA expression of NLS-GFPu and NEDD8 was monitored as described in Methods. Data represent the average +/- SD of 2 independent experiments. Each experiment was performed in triplicates. C) Similar experiment as in (B) but cells were ^{35}S-Methionine pulsed for 20min. Extracts were used for GST pull-down with GST-GFPtrap. Eluates were analysed by SDS-PAGE and gels were dried and exposed to X-ray film.

Supplementary Figure 6

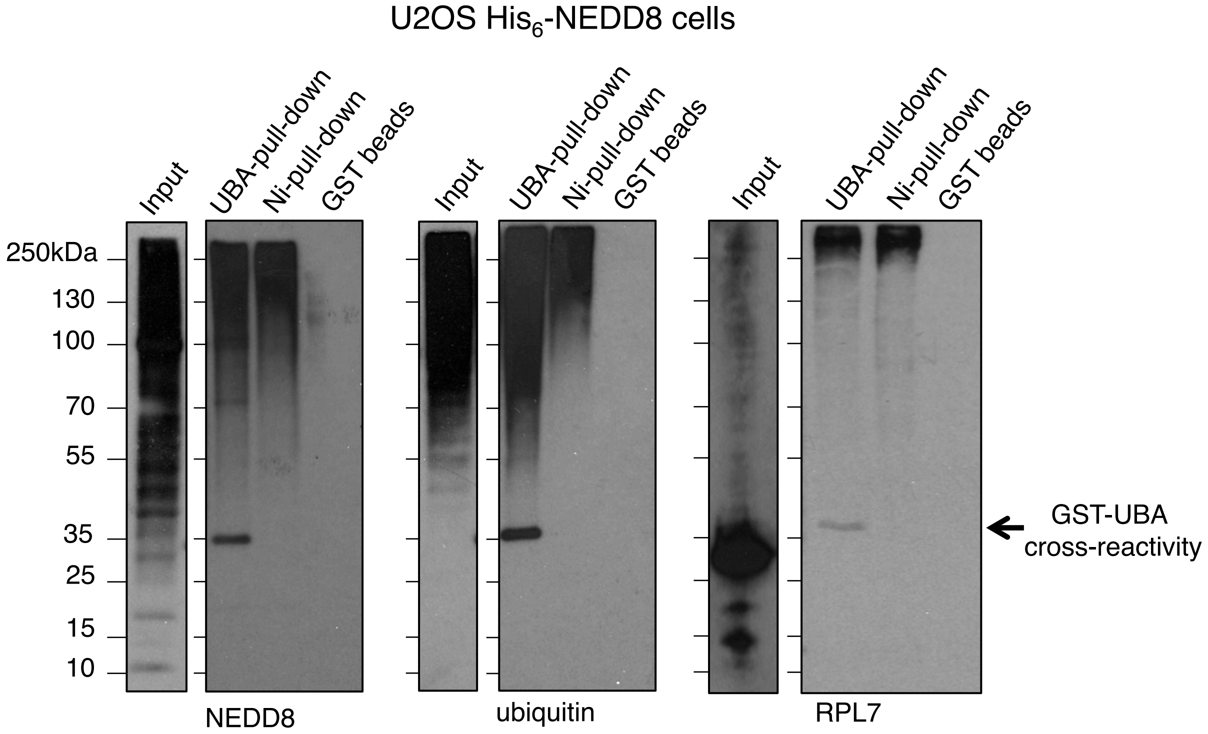

Supplementary Figure 6. RPL7 modification with NEDD8 and ubiquitin upon proteotoxic stress. U2OS cells stably expressing His_6-NEDD8 were treated with MG132 (25μM, 2hrs). Extracts were used for a GST-UBA pull-down. Eluates were then used for a Ni-NTA pull-down. Input and eluates from each purification were analysed by western blotting as indicated.

Supplementary Figure 7

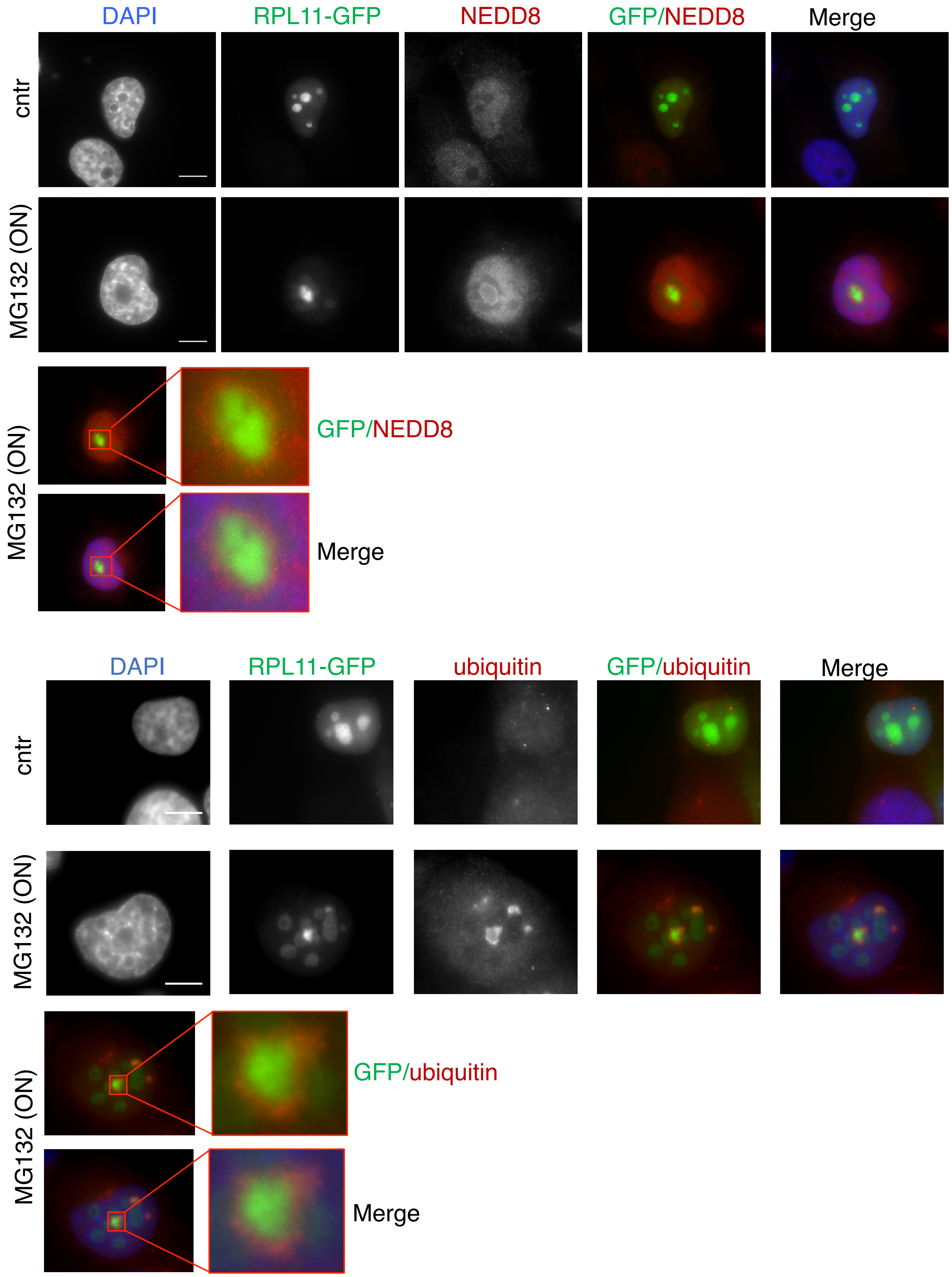

Supplementary Figure 7. RPL11 is localised within the NEDD8/ubiquitin nuclear structures upon severe proteotoxic stress. Experiment performed as in Figure 5A, B, using instead RPL11-GFP. Enlarged insets represent the NEDD8/ubiquitin stained nuclear structures observed upon MG132 surrounding RPL11. Scale bars, 10μm.

Supplementary Figure 8

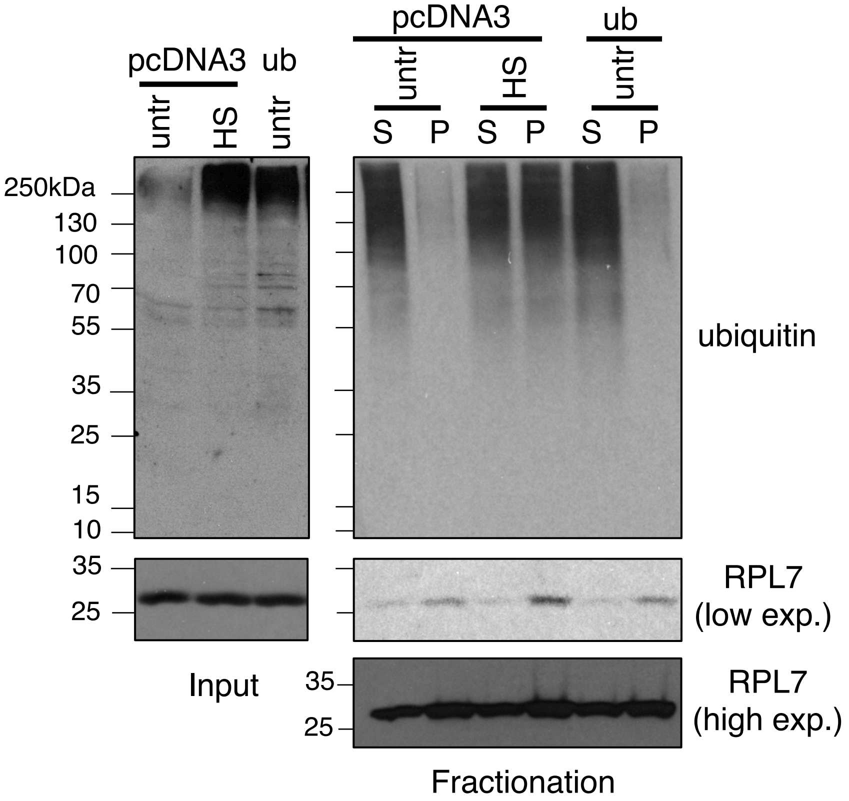

Supplementary Figure 8. Ubiquitin overexpression does not lead to aggregation. Experiment was performed as in Figure 6B, except that 4μg of pcDNA3 ubiquitin expression construct was transfected.

Supplementary Figure 9

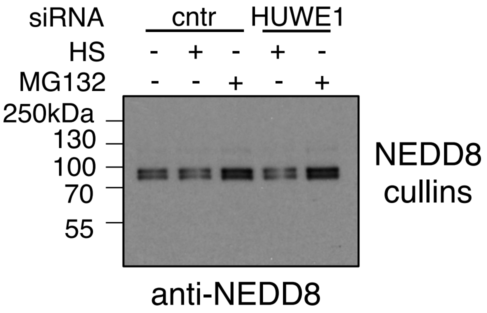

Supplementary Figure 9. A low exposure of western blot analysis for extracts in experiment performed in Figure 7C.

Supplementary Figure 10

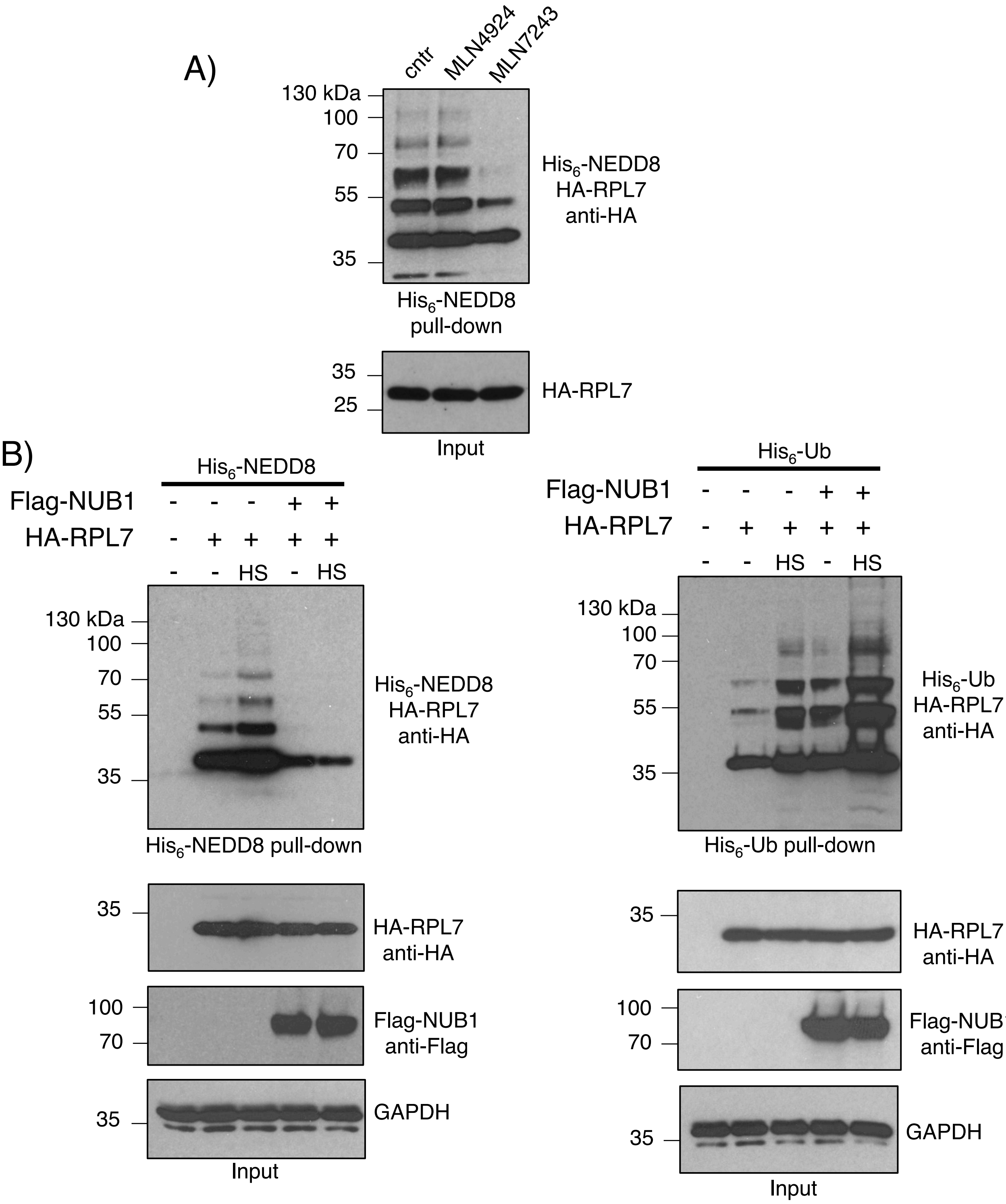

Supplementary Figure 10. NUB1 inhibits UBA1 dependent RPL7-NEDDylation and promotes RPL7-ubiquitination. A) U2OS cells were transfected with 3μg of HA-RPL7 and 4μg of His_6-NEDD8 for 48hrs. Cells were treated 15hrs before harvest with MLN4924 (500nM) and MLN7243 (100nM). His_6-NEDD8 pull-down was performed as described in Methods. B) Transfections were performed as in (A) using 4μg of His_6-NEDD8 or His_6-ubiquitin and 5μg Flag-NUB1 constructs. 48hrs post-transfection cells were heat shocked as indicated for 1hr before lysis and Ni-NTA pull-down. Western blot analysis on the eluates and input was performed with the indicated antibodies.

Supplementary Figure 11

Uncropped Scans

Figure 1

A

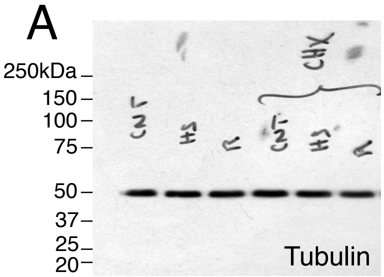

B

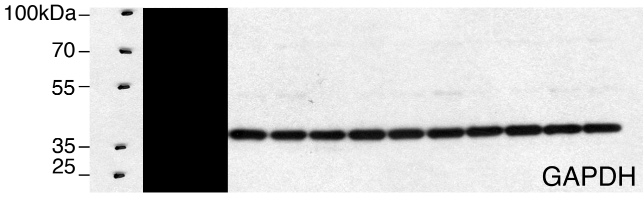

C

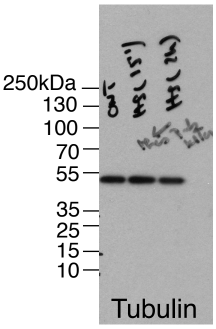

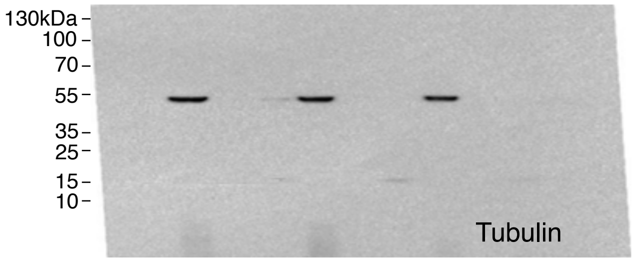

Figure 2

D

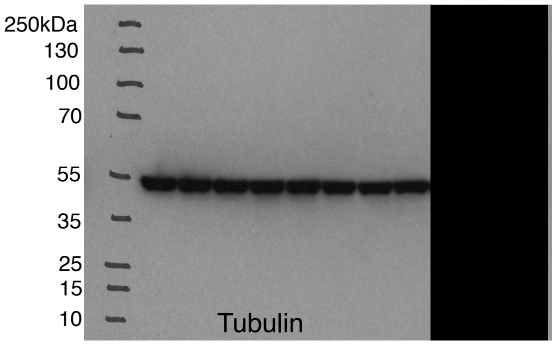

Figure 3

A

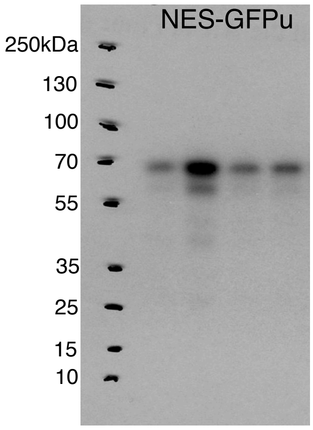

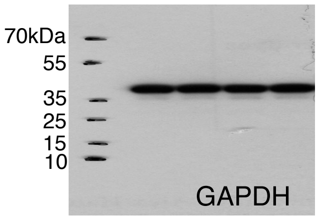

B

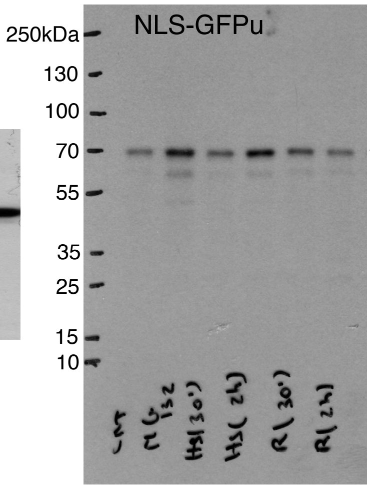

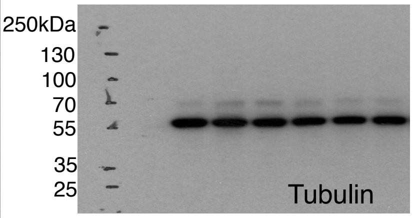

Figure 3

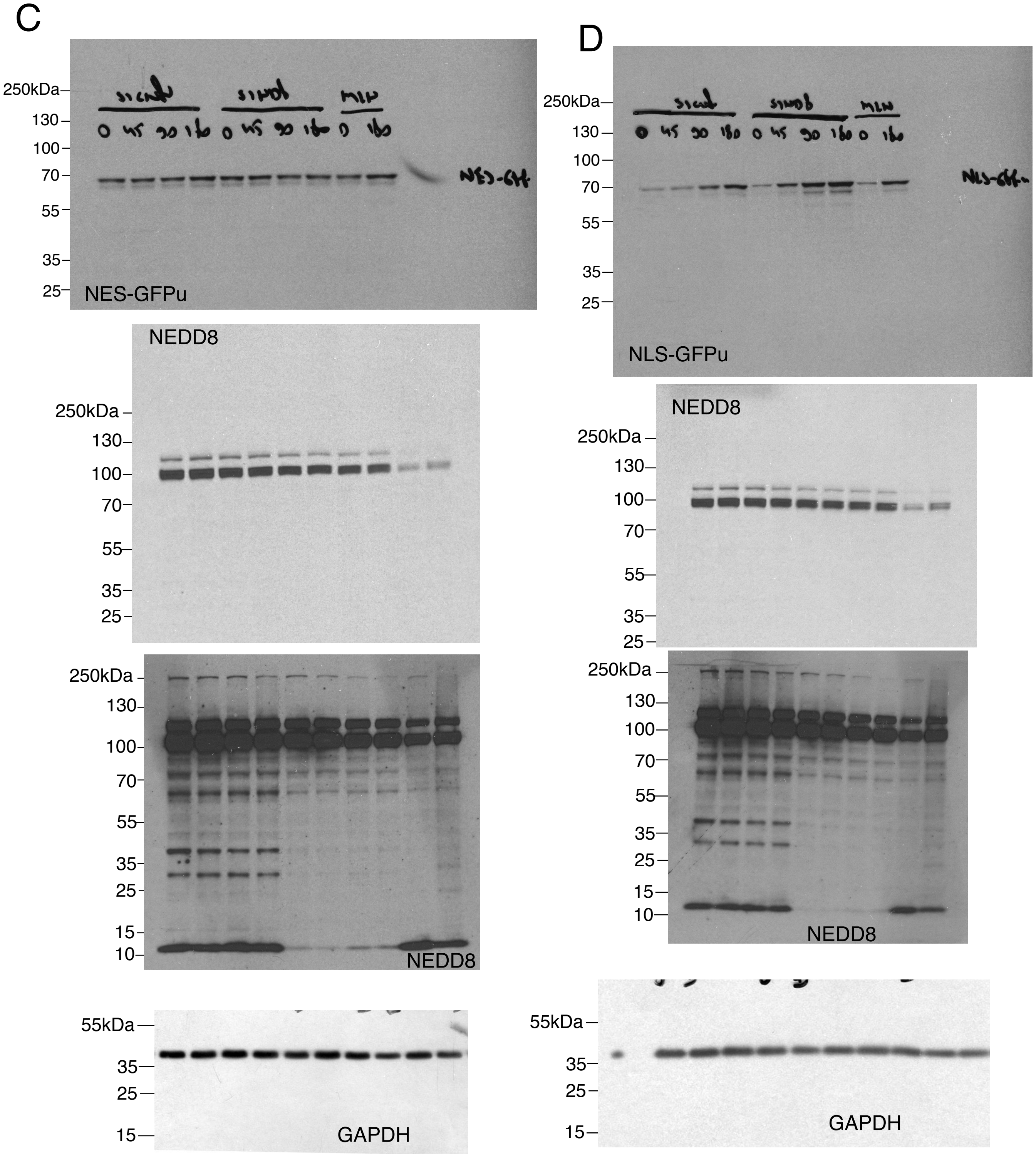
C
D
250kDa
130
100
70
55
35
25
15
10
NES-GFPu
NLS-GFPu
NEDD8
55kDa
GAPDH

Figure 3

E

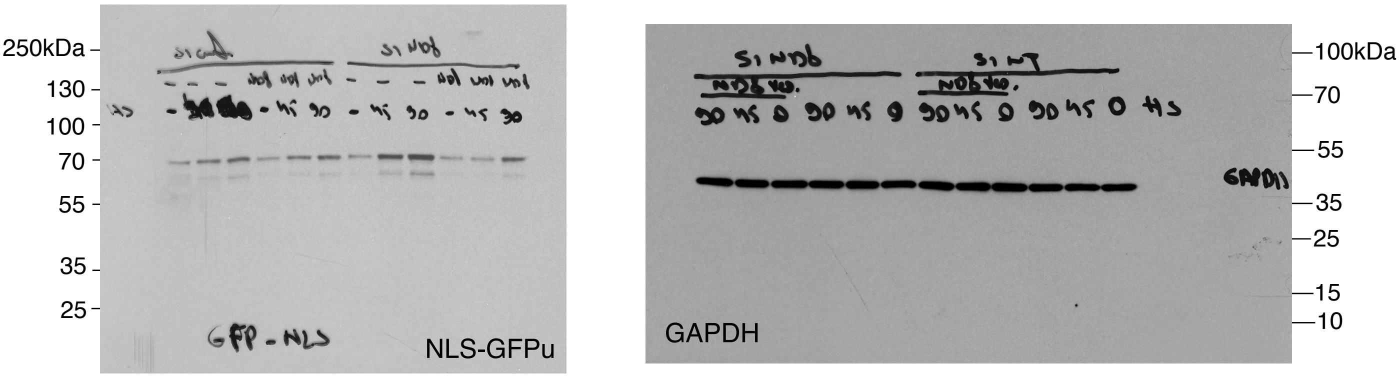
250kDa
130
100
70
55
35
25
NLS-GFPu
GAPDH
100kDa
70
55
35
25
15
10

Figure 4

A

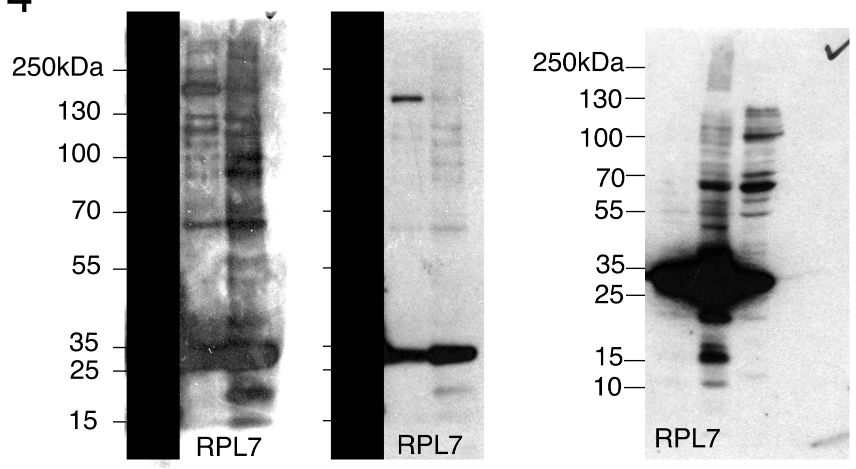

B

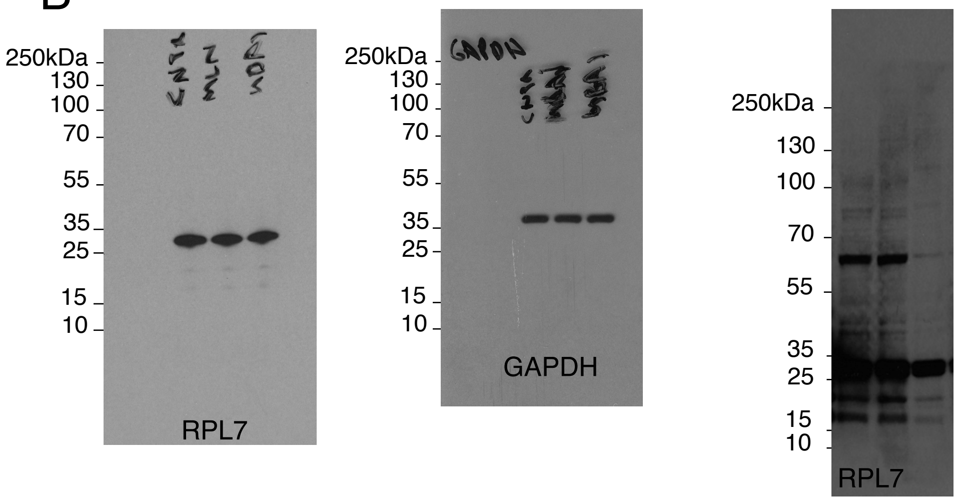

Figure 6

B

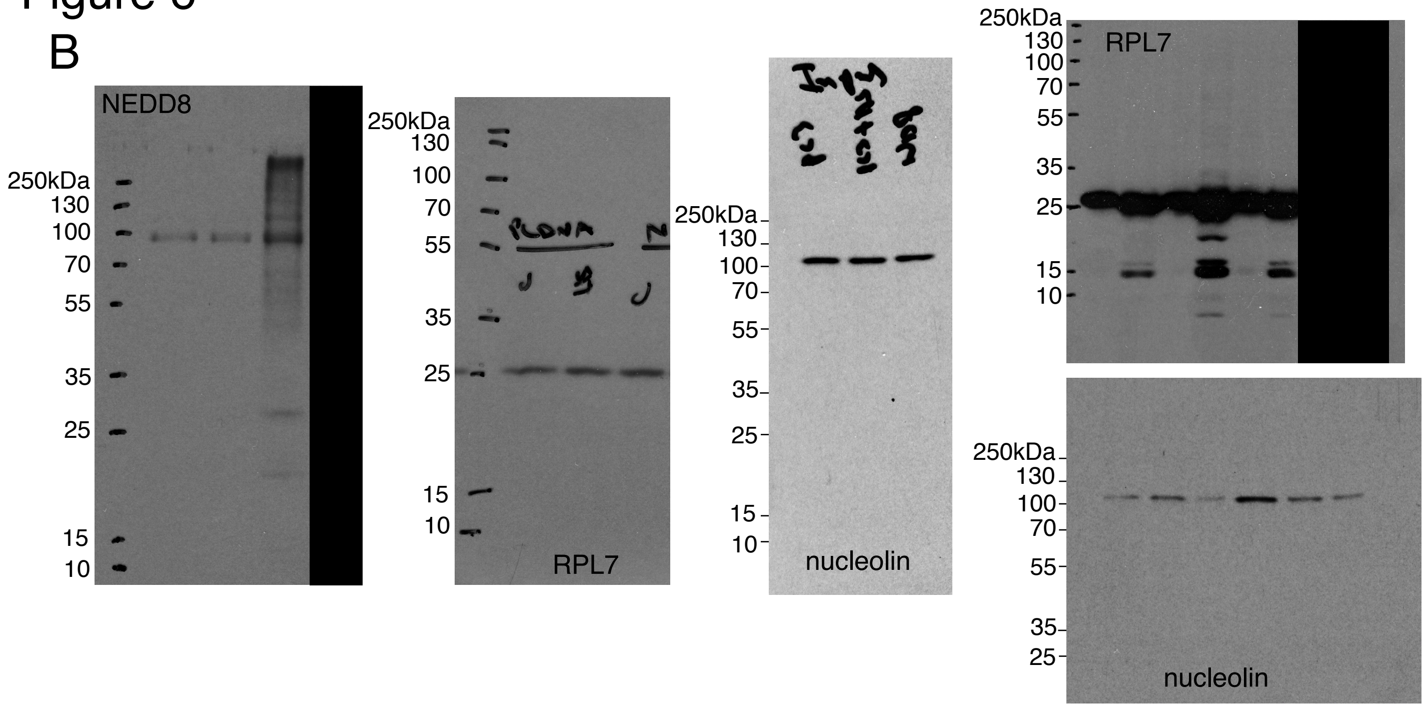

Figure 6

C

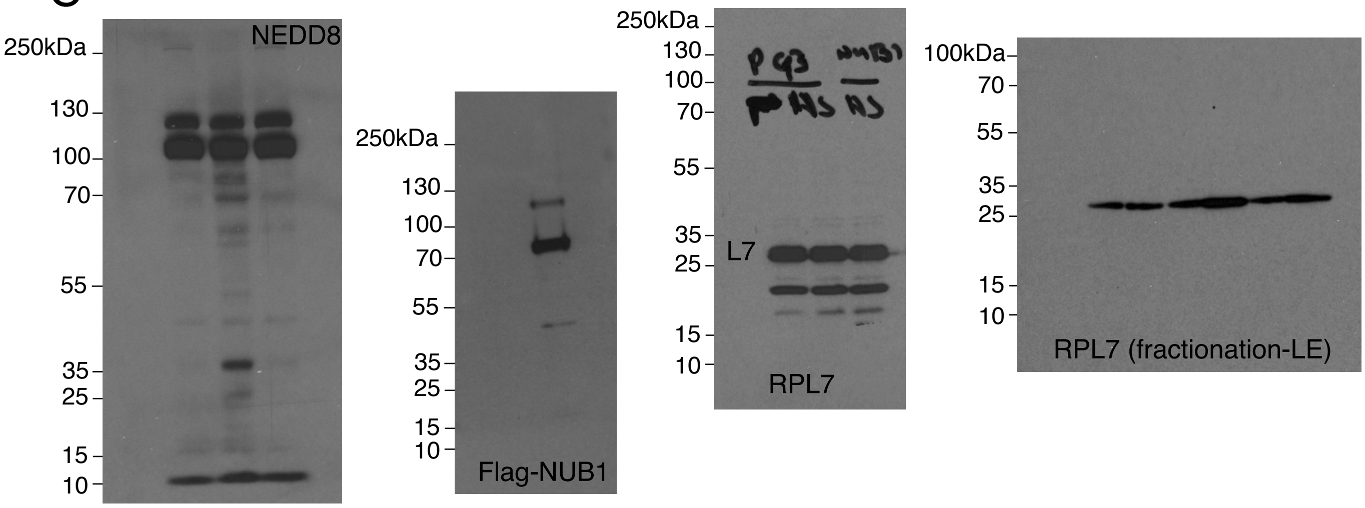

Figure 7

C

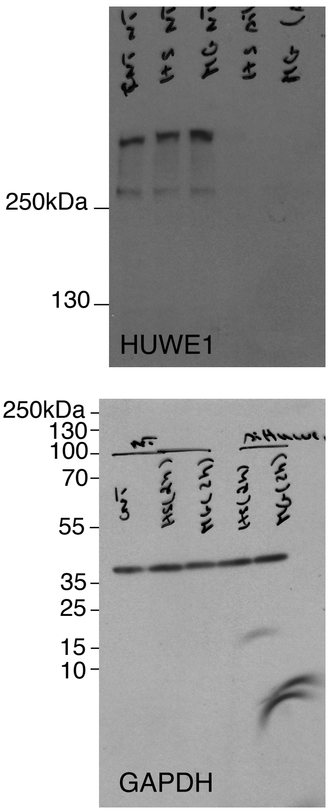

D

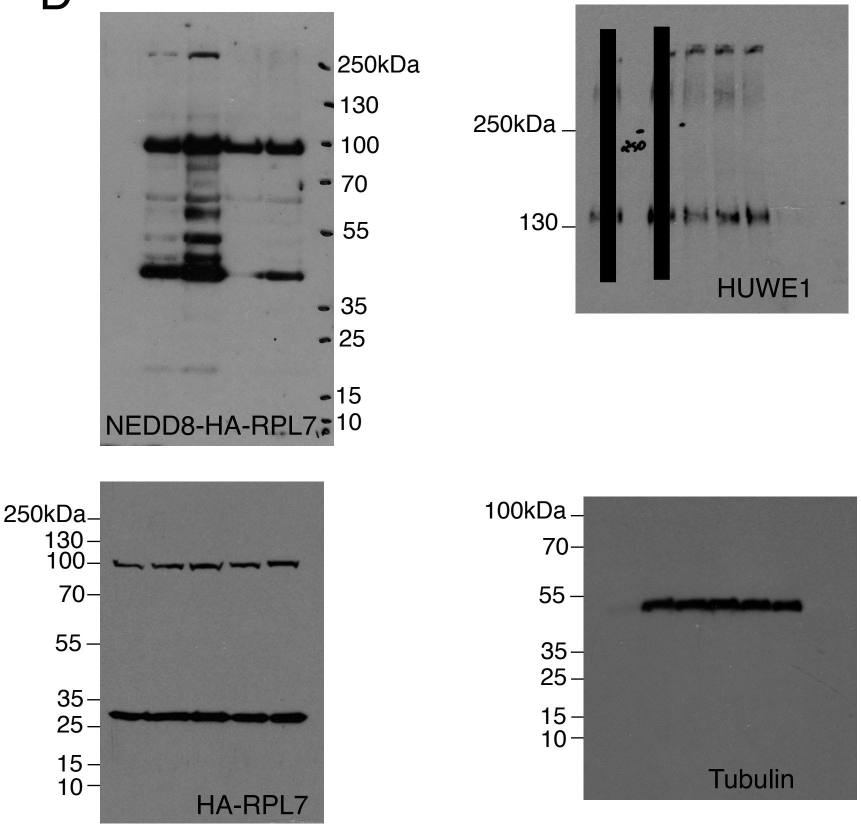

Figure 8

A

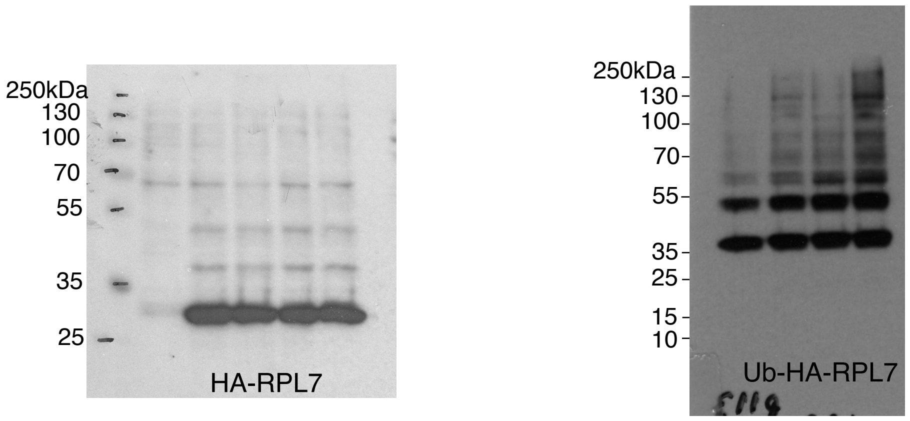
250kDa
130
100
70
55
35
25
HA-RPL7
250kDa
130
100
70
55
35
25
15
10
Ub-HA-RPL7

B

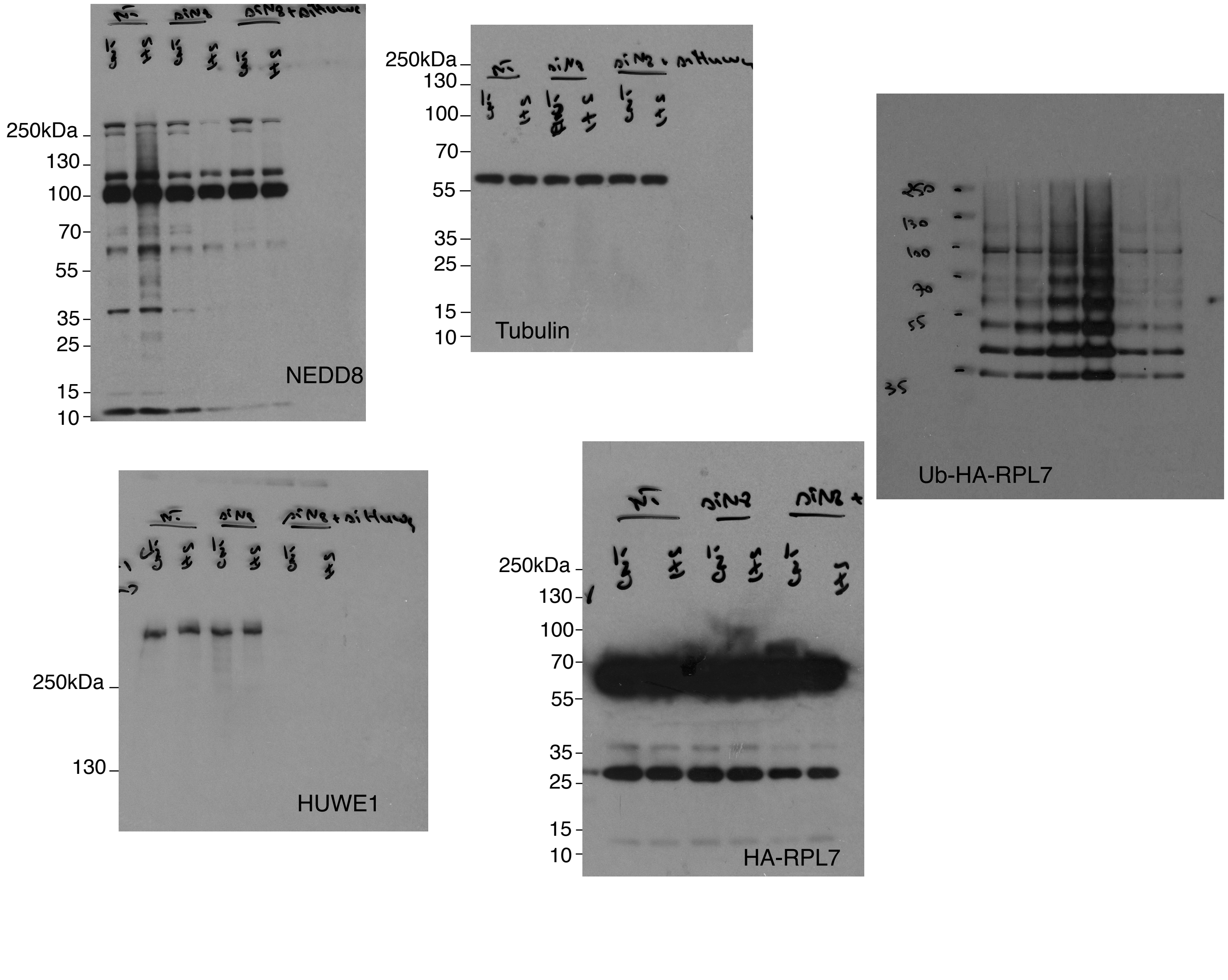
250kDa
130
100
70
55
35
25
15
10
NEDD8
250kDa
130
100
70
55
35
25
15
10
Tubulin
Ub-HA-RPL7
250kDa
130
HUWE1
250kDa
130
100
70
55
35
25
15
10
HA-RPL7

Figure 8

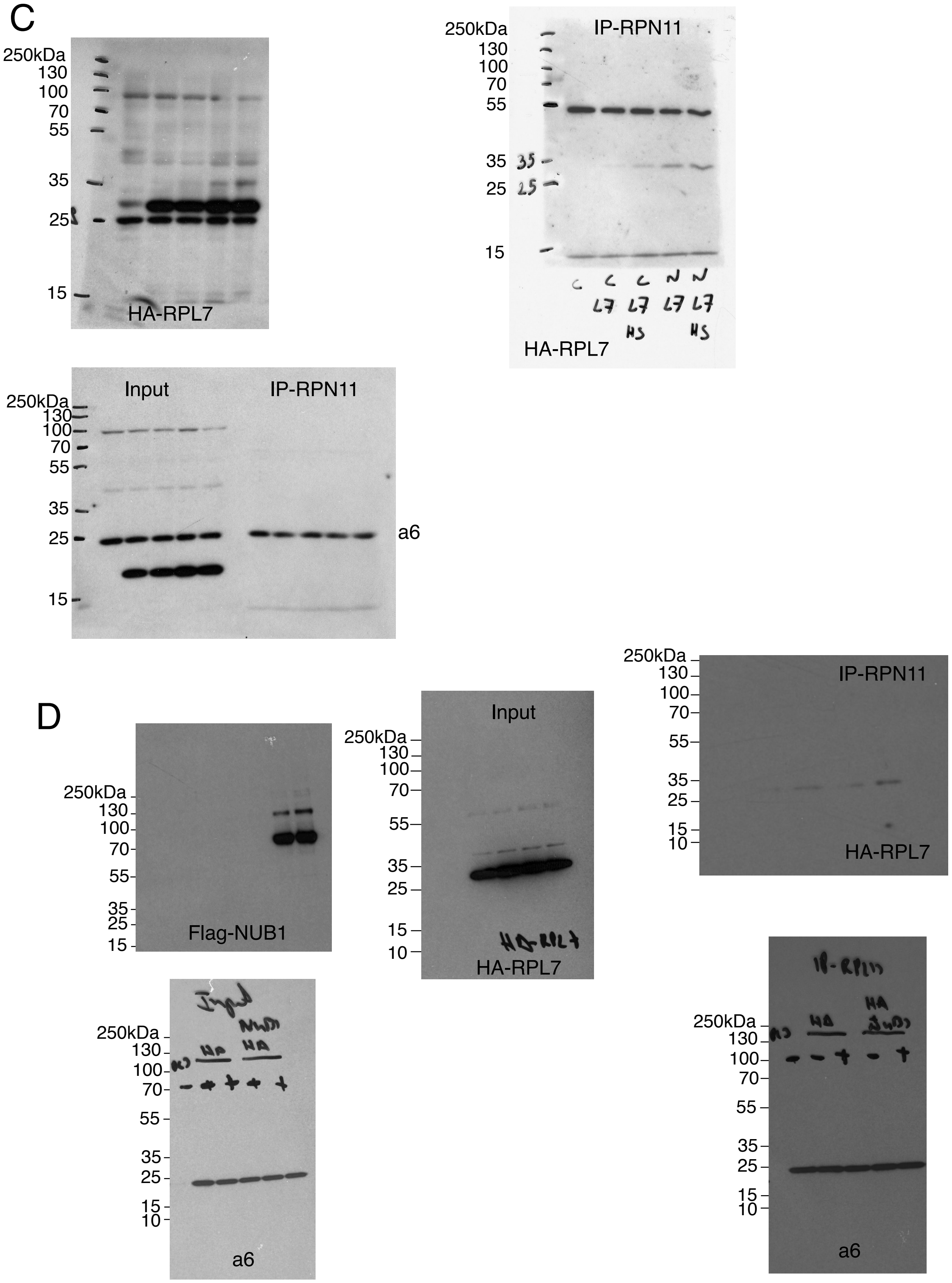
C
250kDa
130
100
70
55
35
25
15
HA-RPL7
IP-RPN11
HA-RPL7
Input
IP-RPN11
a6
D
Flag-NUB1
Input
HA-RPL7
IP-RPN11
HA-RPL7
a6
a6

Figure 9

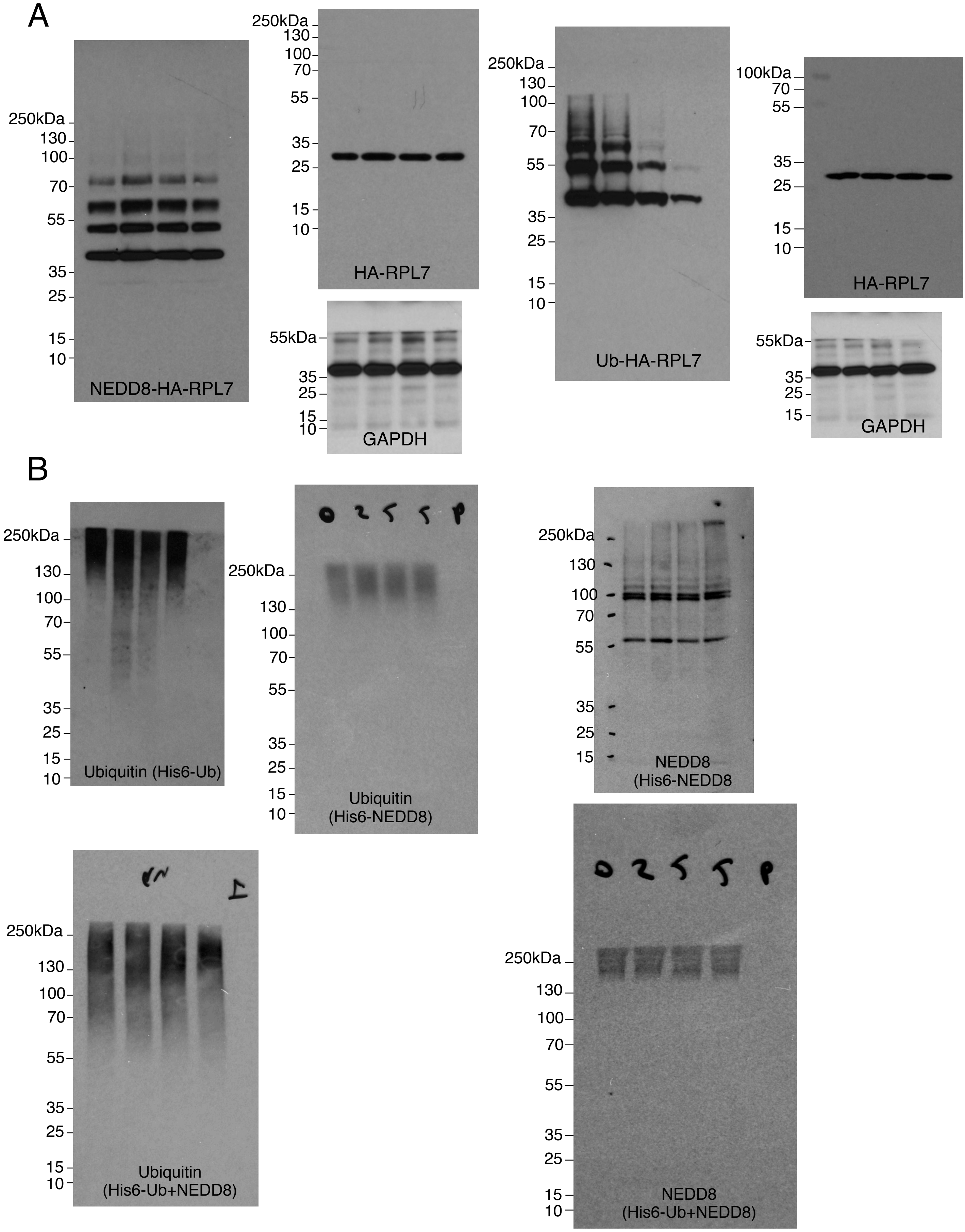
A
NEDD8-HA-RPL7
HA-RPL7
GAPDH
Ub-HA-RPL7
HA-RPL7
GAPDH
250kDa
130
100
70
55
35
25
15
10
B
Ubiquitin (His6-Ub)
Ubiquitin
(His6-NEDD8)
NEDD8
(His6-NEDD8
Ubiquitin
(His6-Ub+NEDD8)
NEDD8
(His6-Ub+NEDD8)

Supplementary Figure 1

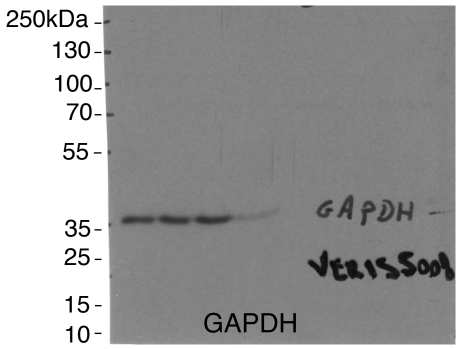

Supplementary Figure 3

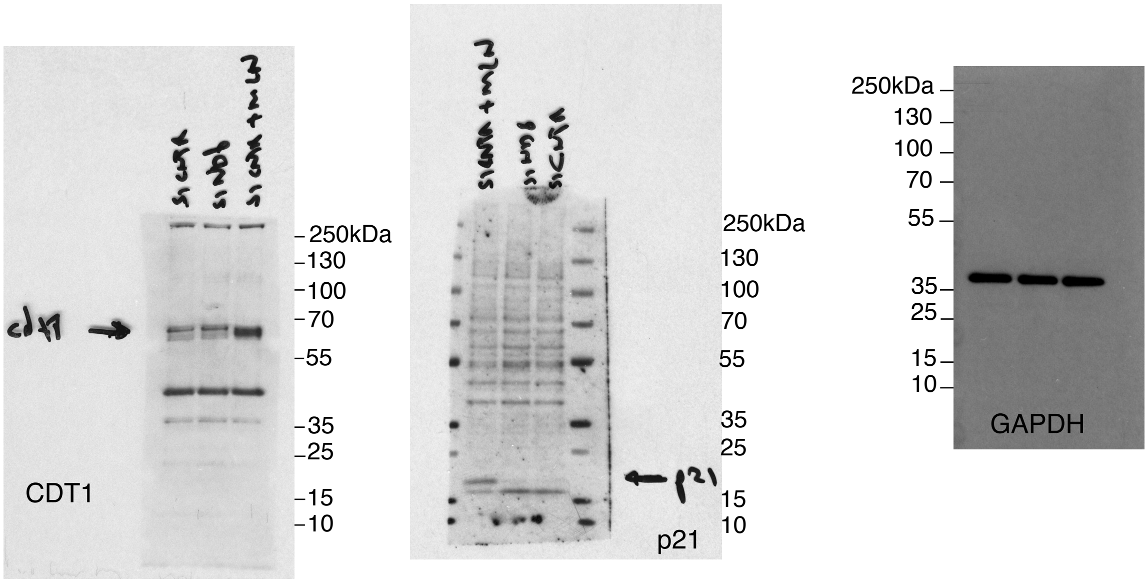

Supplementary Figure 5

A

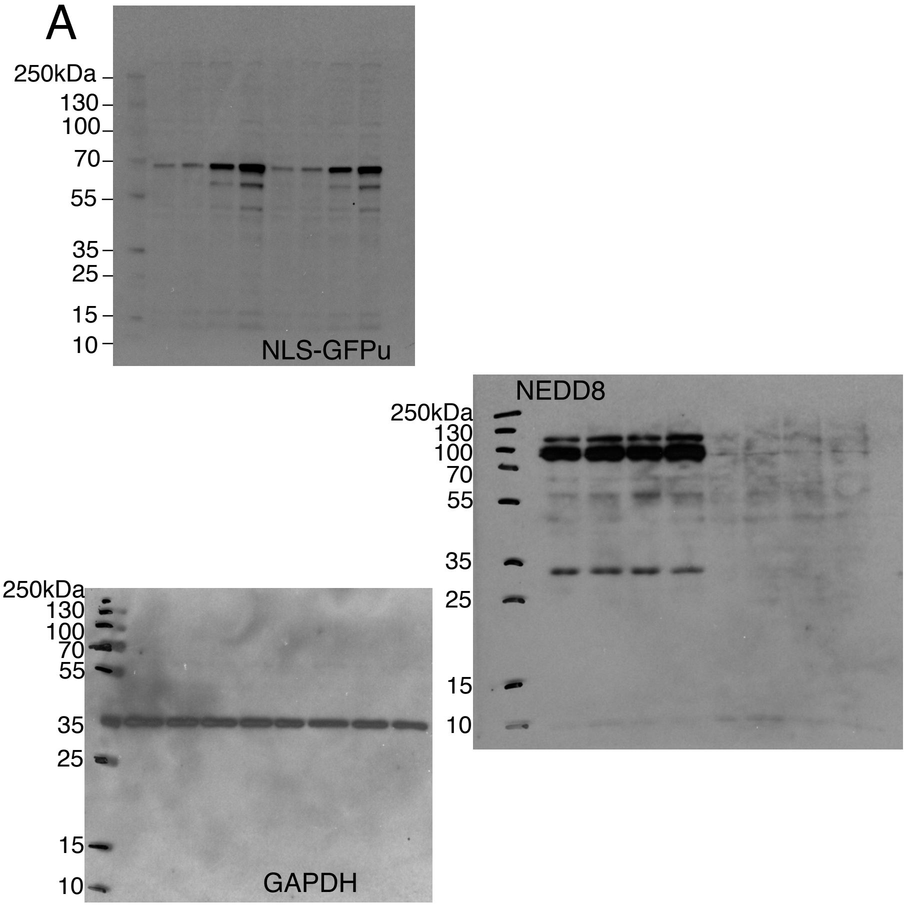

C

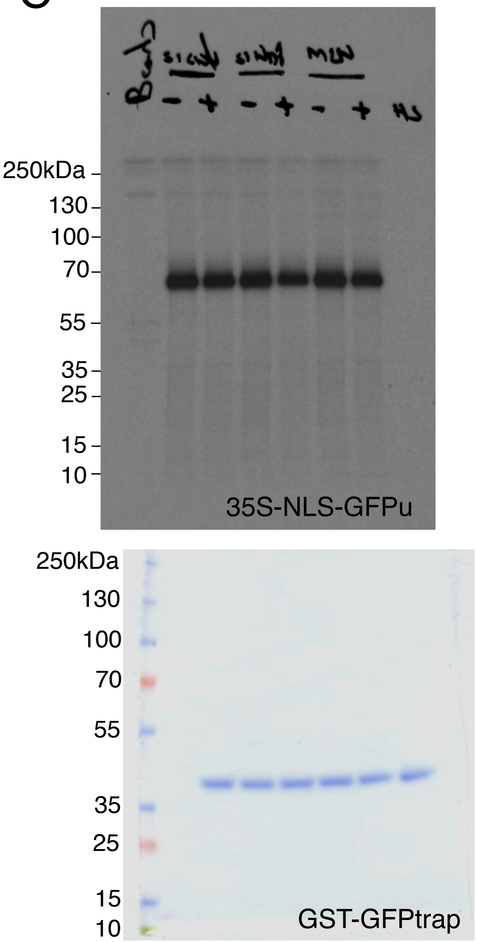

Supplementary Figure 8

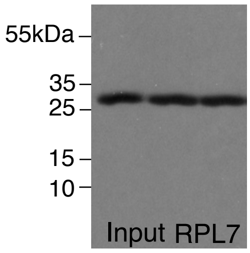

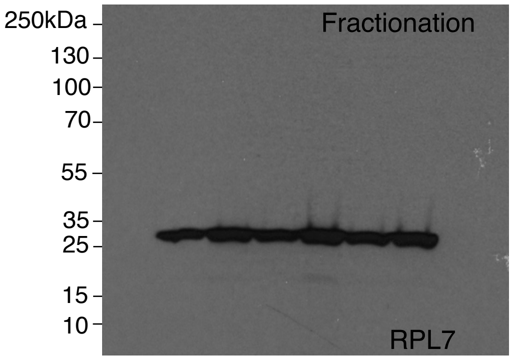

Supplementary Figure 9

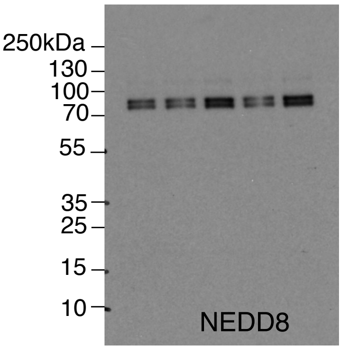

Supplementary Figure 10

A

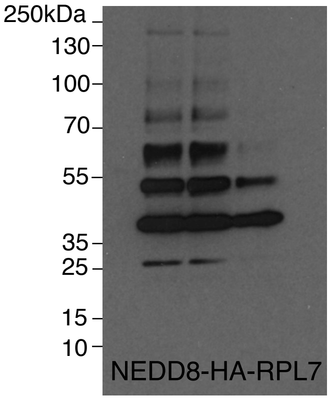

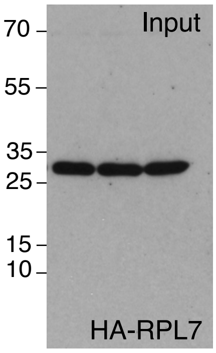

Supplementary Figure 10

B

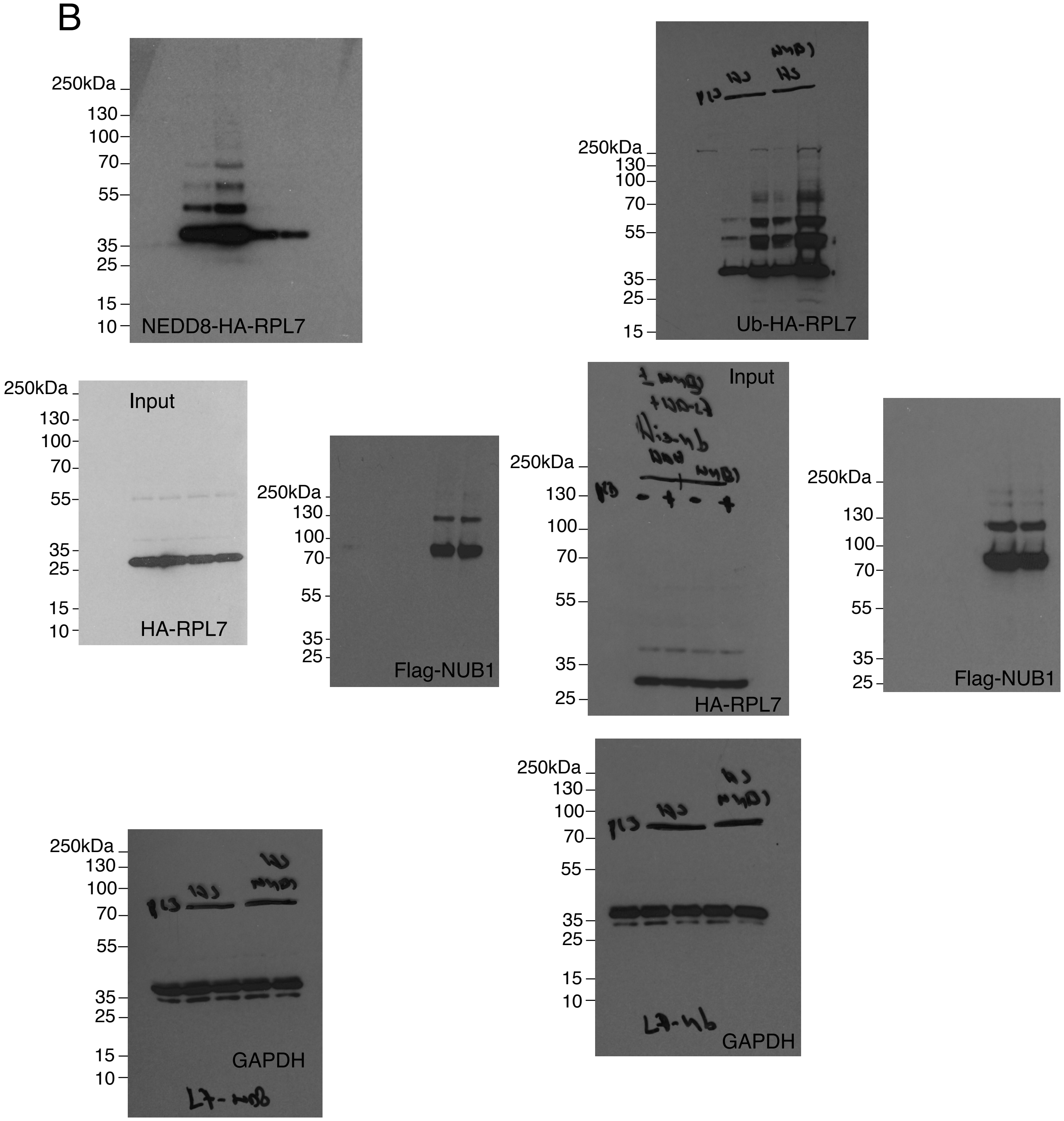

Supplement: Supplementary file 1 — Supplementary Information [file 41467_2018_6365_MOESM1_ESM.pdf]
