# Supplementary material for: NEDDylation promotes nuclear protein aggregation and protects the Ubiquitin Proteasome System upon proteotoxic stress
Source: Nat Commun. 2018 Oct 22;9:4376. doi: 10.1038/s41467-018-06365-0 (PMC6197266; doi:10.1038/s41467-018-06365-0)
Supplement: Supplementary file 3 — Description of Additional Supplementary Files [file 41467_2018_6365_MOESM3_ESM.pdf]

Description of Additional Supplementary Files

File Name: Supplementary Data 1

Description: Effect of siNEDD8 and MLN4924 on the composition of heat-shock induced aggregates analyzed by SILAC mass spectrometry-based proteomics.
